# Filtering, FDR and power

**DOI:** 10.1186/1471-2105-11-450

**Published:** 2010-09-07

**Authors:** Maarten van Iterson, Judith M Boer, Renée X Menezes

**Affiliations:** 1 Center for Human and Clinical Genetics, Leiden University Medical Center, Postzone S4-P, P.O. Box 9600 Leiden, 2300 RC, The Netherlands; 2 Netherlands Bioinformatics Centre, P.O. Box 9101, Nijmegen, 6500 HB, The Netherlands; 3 Department of Epidemiology and Biostatistics, VU University Medical Center, PK 6Z 183, P.O. Box 7057, Amsterdam, 1007 MB, The Netherlands; 4 Laboratory of Pediatrics, Erasmus Medical Center/Sophia Children's Hospital, P.O. Box 2060, Rotterdam, 3000 CB, The Netherlands; 5 Centre for Medical Systems Biology, P.O. Box 9600, Leiden, 2300 RC, The Netherlands

## Abstract

**Background:**

In high-dimensional data analysis such as differential gene expression analysis, people often use filtering methods like fold-change or variance filters in an attempt to reduce the multiple testing penalty and improve power. However, filtering may introduce a bias on the multiple testing correction. The precise amount of bias depends on many quantities, such as fraction of probes filtered out, filter statistic and test statistic used.

**Results:**

We show that a biased multiple testing correction results if non-differentially expressed probes are not filtered out with equal probability from the entire range of p-values. We illustrate our results using both a simulation study and an experimental dataset, where the FDR is shown to be biased mostly by filters that are associated with the hypothesis being tested, such as the fold change. Filters that induce little bias on the FDR yield less additional power of detecting differentially expressed genes. Finally, we propose a statistical test that can be used in practice to determine whether any chosen filter introduces bias on the FDR estimate used, given a general experimental setup.

**Conclusions:**

Filtering out of probes must be used with care as it may bias the multiple testing correction. Researchers can use our test for FDR bias to guide their choice of filter and amount of filtering in practice.

## Background

Statistical analysis of high dimensional data, i.e. those for which the number of parameters *p *is much larger than the number of samples *m*, often involves testing of multiple hypotheses. This is because models typically associate one parameter to each feature on the microarray used, which may represent a part of or a whole gene (in case of cDNA arrays and of oligonucleotide arrays in general) or any genomic section. So if classic statistical methods are used to analyse the data per feature, computed p-values must be jointly corrected for multiple testing [1]. Of course, the larger the number of hypotheses tested, the stronger the correction for multiple testing must be in order to keep the error rate acceptably low.

To decrease the penalty incurred by multiple testing correction, some articles (see for example McCarthy and Smyth [2], Zhang and Cao [3] and references therein) make a selection of features prior to the data analysis that, it is hoped, are more likely to not conform with the null hypothesis. For clarity, we call such features non-null, in contrast with those that follow the null hypothesis which we call null features. By having a weaker correction for multiple testing, it is also hoped to improve power. However, such selection may have undue effect on results. Firstly, by leaving some features out of the analysis altogether, some non-null features will also be left out, therefore putting a bound on potential power. Secondly, it is impossible to select only features that do not follow the null hypothesis - had we known which ones these were, we would not have needed testing in the first place. So many features, including some null, are left in, and as such multiple testing correction must still be used. However, commonly used multiple testing correction methods rely on the assumption that the null p-values follow a uniform distribution, which may no longer be the case amongst the selected features. This may introduce bias on the corrected p-values.

The effect of feature selection on power to detect probes that are differentially expressed between two groups can also be assessed. Feature selection yields an increase of the p-value significance threshold and, as a consequence, the power is largely expected to increase too. However, as we will show this is not always the case.

Thus feature selection methods may affect both power and overall error rate estimation. However, neither can be evaluated in practice, as they require knowledge of which features conform (or not) with the null hypothesis. So it is not straightforward to know in practice the exact effect of feature selection. In this paper we shall evaluate the effects of feature selection procedures, first theoretically and subsequently illustrated by both a simulation study and publicly available experimental data. These selection procedures are often referred to as "filters", because they are meant to "filter out" some of the noise (null features) of the data. We shall consider multiple testing correction methods that estimate the false discovery rate (FDR), under the assumption that null p-values follow a uniform distribution (for other FDR estimation methods see Discussion and Additional File 1). We shall describe common types of filter used in the Methods section, and subsequently study their impact on both overall estimated error rates and power.

## Methods

### Filtering violates FDR methods' assumption

Here we assume a study setup commonly found in practice, involving gene expression profiles of two groups of independent samples, with the null hypothesis *H*_0*i *_: *μ_iA _*= *μ_iB _*representing no differential expression between the two groups *A *and *B*, for any given gene *i*, and a corresponding two-sided alternative hypothesis *H_ai _*: *μ_iA _*≠ *μ_iB_*. Let {*V_i_*}, {*R_i_*}, *i *= 1, ..., *m *be sets of binary variables taking values in {0, 1}, such that *V_i _*= 1 if gene *i *follows *H*_0_, and *R_i _*= 1 if gene *i *is left in the data after filtering. Let us also consider the filter statistic *W *= *W *(*Z*), so that gene *i *is filtered whenever *W *(*Z_i_*) ≥ *w *for a chosen value *w*, where *Z *represents a test statistic. Then we can write *R_i _*= *I*{*W *(*Z_i_*) ≥ *w*} for all *i*. For more details about this setup, see section "Study design" in Additional File 1.

In practice, multiple testing correction still needs to be used after filtering. Methods that aim at handling the FDR typically assume that, under *H*_0_, the p-values yielded by the statistical test satisfy P~U[0,1] in general, so multiple testing correction after filtering requires that their null distribution, represented by $G_{0}$, after filtering is represented by $G_{0}^{W}$ and also satisfies $G_{0}^{W}=U[0,\;1]$ for a given filter statistic *W*. Its cumulative distribution function (cdf) is

(1) $$\begin{array}{l} G_{0}^{W}(u)=\mathrm{Pr}\left\{P_{i}^{W}\leq u|H_{0}\right\}\\ \;\;\;\;\;\;\;\;\;\;\;\;\;\;=\mathrm{Pr}\left\{P_{i}\leq u|V_{i}=1,R_{i}=1\right\}, \end{array}$$

thus the equality $G_{0}^{W}(u)=G_{0}(u)$ for all *u*, holds if, and only if,

(2) $$\mathrm{Pr}\{P_{i}\leq u|V_{i}=1,\;R_{i}=1\}=\mathrm{Pr}\{P_{i}\leq u|V_{i}=1\}$$

which means that *R_i _*must be independent of *V_i _*and of *P_i_*. In other words, if the filter selects null features from the entire range [0,1] with equal probability, then the null distribution of the p-values remains U[0,1]. This assumption is notably difficult to check, as it is not known which of the remaining p-values follow  U[0,1]. Note also that this is not required from alternative features.

### Filter statistics

Various criteria are used by researchers to filter features out of a dataset. We aim at evaluating filter effects on error estimates and power and, as such, will consider a few filter types used in practice, but these are not intended to cover all possible filters.

A commonly used method involves leaving out of the dataset features with measurement very close to, or less than, background. We shall refer to this as the *signal *filter, and we base it on the average signal observed for the feature over the two groups, i.e. $W_{S}(Z)=(\bar{X}+\bar{Y})/2$. A second type of filter commonly used is based on the absolute value of the (log) fold change, i.e. $W_{FC}(Z)=\;|\bar{X}-\bar{Y}|$. It aims at leaving out of the analysis features with too small a fold change to be biologically interesting, and we shall refer to it as the *fold change *filter. A third type of filter of practical interest leaves out of the analysis features that overall vary less than a certain given threshold. This *variance *filter assumes that the feature-specific variance reflects how much discrimination that feature may yield between the groups, and we shall express it as $W_{V}(Z)=S_{Z}^{2}$.

The aforementioned statistics will be used to filter out uninformative features here.

### Effect of filters on multiple testing correction

Multiple testing correction can be done in a variety of ways. Essentially, methods aim at controlling/estimating either one of two different error types, namely family-wise error rate (FWER) and false discovery rate (FDR). In most gene expression data analysis applications, it is of interest to handle the FDR, as it makes more sense to talk about the proportion of false positives in a list of genes declared differentially expressed, instead of the probability of making at least one mistake in all tests. For this reason, we focus on methods that aim at handling the FDR, most of which assume that null p-values follow a U[0, 1], so can be biased by the use of a filter that affects the validity of this assumption.

Thresholds for significance yielded by multiple testing methods increase as the number of hypotheses tested *m *decreases. For example, consider the original Benjamini and Hochberg [1] step-up procedure for (strong) control of the FDR for independent test statistics, which can be described as follows. In order to control the FDR at level *ϕ*, reject the null hypothesis *H*_0*i *_whenever the p-value *P_i _*is no greater than (*iϕ*)/*m *for each *i *= 1, ..., *m*. When some filter is applied to the data, resulting in *γm *features retained for further analysis, the new FDR threshold becomes (*iϕ*)/(*γm*), which is larger than the one for all features as 0 <*γ *< 1. This suggests that an improved power may result from filtering, as a larger threshold may select more truly differentially expressed genes. However, the actual effect on power and on achieved FDR depends on the filter statistic used. To consider those analytically it is convenient to write the multiple testing procedure as an explicit function of the empirical cdf of the p-values.

The commonly used FDR-controlling multiple-testing procedures suggested by Y. Benjamini and co-authors can all be expressed in terms of the empirical cdf of the p-values *G_m _*[4]. Indeed, consider the general procedure of selecting p-values *P_i _*satisfying *P_i _*≤ u*, where

(3) $$u*=\underset{u}{\max}\{g(u_{i},\phi )\leq G_{m}(u),\;0\leq u\leq 1\},$$

and *u *represents possible values for the random variables {*P_i_*} (*i *= 1, ..., *m*). Then different functional forms of *g*(*u_i_*, *ϕ*) will yield the different FDR methods: Benjamini and Hochberg [1]'s step-up procedure uses *g*(*u*, *ϕ*) = *u/ϕ *, the adaptive FDR of Benjamini *et al. *[5], which corrects for the proportion of null features *π*_0_, uses *g*(*u*, *ϕ*) = (*π*_0_)*u/ϕ *, both independent of *i*, and Benjamini and Yekutiely [6]'s method uses $g(u_{i},\;\;\phi )=u/\phi \sum_{j}^{i}1/j$. For more details see section "FDR methods and p-values distributions" in Additional File 1.

The effect of filtering on power can then be evaluated by using the relation *G_m_*(*u*) = *π*_0_*G*_0 _- (1 - *π*_0_)*G_a_*(*u*). Doing so the expression on the right-hand side of 3 becomes max*_u_*{[*g*(*u_i_*, *ϕ*) - *π*_0_*G*_0_(*u*)]/(1 - *π*_0_) ≤ *G_a _*(*u*), 0 ≤ *u *≤ 1}. For example, if after filtering the original Benjamini-Hochberg FDR is to be used, the power is given graphically by the intersection $G_{a}^{W}(u)=[1/(1-\pi_{0}^{W})][u/\phi -\pi_{0}^{W}G_{0}^{W}(u)]$, where $\pi_{0}^{W}$ represents the proportion of null features after filtering (see [7] when no filtering is applied).

For one example of such a development, see section "FDR and power as function of fraction filtered out" in Additional File 1.

For some methods it is not possible to express them as explicit functions of the p-values' cdf. Then numerical methods can be used to evaluated the effect of filtering, as done in our simulation study in section "Simulation study".

### Student's t test

To further evaluate the effect of filtering, null and alternative distributions of the test statistic, before and after filtering, must be known. A commonly used statistic in the study setup used here to test the null hypothesis *H*_0 _: *μ*_*X*_ = *μ*_*Y*_against *H_a _*: *μ*_*X*_≠ *μ*_*Y*_ is

(4) $$T=\frac{{\bar{X}}_{nX}-{\bar{Y}}_{nY}}{{\left[\hat{\text{V}}\text{ar}\left({\bar{X}}_{nX}-{\bar{Y}}_{nY}\right)\right]}^{1/2}}=\frac{{\bar{X}}_{nX}-{\bar{Y}}_{nY}}{S_{p}\sqrt{1/n_{X}+1/n_{Y}}},$$

where we assume for simplicity that $\sigma_{X}^{2}=\sigma_{Y}^{2}=\sigma^{2}$, so $S_{p}^{2}$ represents the pooled variance. Under *H*_0 _the distribution of *T *is a Student's t distribution with *v *= *n_X _*+ *n_Y _*- 2 degrees of freedom.

The effect of filtering can be evaluated via conditioning on the filter statistics. For example, if a fold change filter is used, the conditional cdf can be written as

(5) $$\begin{array}{l} F_{T_{W}}(t)=Pr\{T\leq t|\;\;\;|{\bar{X}}_{n_{X}}-{\bar{Y}}_{n_{Y}}|\;\;\;\geq w\}\\ \;\;\;\;\;\;\;\;\;\;\;\;\;\;=\frac{Pr\{T\leq t,|{\bar{X}}_{n_{X}}-{\bar{Y}}_{n_{Y}}|\;\;\geq w\}}{Pr\{|{\bar{X}}_{n_{X}}-{\bar{Y}}_{n_{Y}}|\;\;\geq w\}}. \end{array}$$

Similar expressions can be derived for the other filter statistics (see section "Density of test statistics after filtering" in Additional File 1).

Once an expression for the pdf of the test statistics after filtering is obtained, we can obtain the pdf and cdf of the p-values using the relation *P *= 2[1 - *F*_0_(|*T*|)], which holds since *P *= *P*{*T *> |*t*_0_|} = 1 - *F*(|*t*_0_|) + *F*(-|*t*_0_|) and *F *is assumed to be symmetric (see also section "Distribution of p-values" in Additional File 1). Similar relationships can also be obtained for non-symmetric *F*. For expressions corresponding to some of the filter statistics, see section "Filtering and p-values distribution" in Additional File 1.

**Figure 1:**
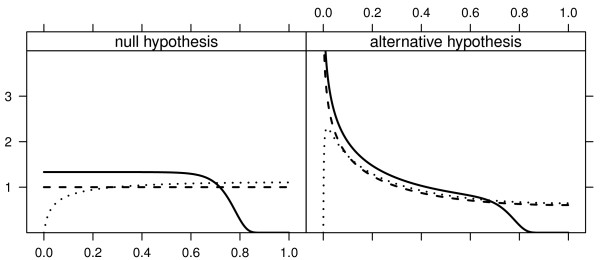
**Probability density functions (pdf) of p-values for two filters under the null hypothesis (*t*_*v *= 8 _(δ = 0)) (left panel) or alternative hypothesis (*t*_*v *= 8 _(δ = 1)) (right panel)**. For each filter 25% of the hypotheses are removed. The fold-change filter is shown as a solid line, the variance filter as a dashed line, and the pdf with no filtering is shown as a dashed line. For more details on how these were obtained see section "Filtering and p-values distribution" of Additional File 1.

Based upon such expressions obtained with the various filter statistics, we display the effect of each filter on the null and alternative distributions of p-values on figure 1. From it we can see that the fold change filter leaves out mostly features with p-values near 1, whilst the variance filter leaves out more p-values near 0, suggesting the latter is more likely to leave out non-null features.

### A test for filtering-induced FDR bias

Since the bias on the FDR is yielded by the effect of a filter *W *on the null p-values distribution *G*_0_, we propose to compare an estimate of the distribution $G_{0}^{W}$ to the expected uniform. In the setup used here, $G_{0}^{W}$ can be estimated by performing the same statistical analysis on the dataset where row and column labels are permuted, and subsequently applying the filter *W*. Then ${\hat{G}}_{0}^{W}$ can be compared to the uniform using a Kolmogorov-Smirnov test, yielding a p-value *q*. In fact, we use a Benjamini-Hochberg FDR-correction for the p-values, that is equivalent to a one-sided Kolmogorov-Smirnov and thus will be less conservative. This process can be repeated a number *N*_0 _of times, so yielding an empirical p-value distribution, $G_{q}$ say, for the comparison between the filtered null p-value distribution and the U[0,1]. By comparing $G_{q}$ to the uniform using again a Kolmogorov-Smirnov test, it can be concluded whether filtering affects the null p-value distribution and, as such, FDR estimates. If so, researchers may wish to either consider other types of filter, or avoid using a filter altogether. For more details, see section of the same name in Additional File 1.

### Simulation study

#### Study setup

To investigate the properties of the filters described in subsection "Filter statistics" a simulation study is carried out. We use a setup that mimics a microarray experiment where thousands of features are measured simultaneously, based upon a setup first suggested by Langaas *et al. *[8] and described in detail in Additional File 1. Briefly, per feature a two-sample Student-t test statistic was calculated and converted to a two-sided p-value accordingly. The p-value list was then filtered using each one of the filter statistics considered here, and subsequently FDR-corrected by either one of the following methods: Benjamini-Hochberg [1], Benjamini-Yekutieli [6], adaptive Benjamini-Hochberg [5] and Storey [9], represented respectively by BH, BY, aBH and qv. To guarantee comparability, the same fraction (1 - *γ*) of p-values was removed in all cases. For each simulated list, features are declared differentially expressed yielding an FDR of 5%, and subsequently both the achieved FDR (fraction of false positives amongst features below FDR threshold) and the observed power (fraction of p-values below FDR threshold amongst those belonging to non-null features) were calculated.

Note that FDR estimation using the qvalue method relies on p-values taking values on the entire [0,1] interval. Thus, we only used the variance and signal filter statistics with this method.

#### Illustration of filter statistics

We shall show how each filter statistic affects power and FDR estimates using one of the simulated datasets chosen at random. First, the filter statistics used have little association with each other, as evidenced by the lack of pattern described by the dot clouds (Additional File 1, figure S1). It is also clear that, when using any of these filter statistics, there is always a fraction of the truly differentially expressed features that is wrongly left out, which can never be declared differentially expressed.

**Figure 2:**
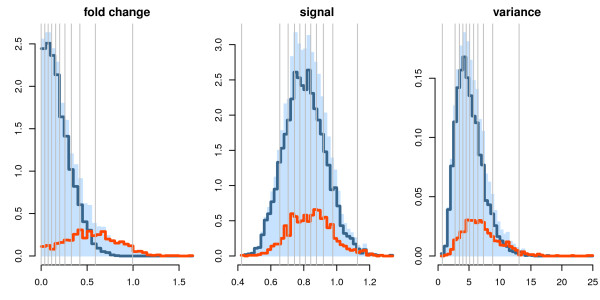
**Filter statistic distribution for all features (blue histogram) and separately for features for which *H*_0 _holds (blue line) and *H_a _*holds (red line) for one simulated dataset**. Each one of these filter statistics leaves features out with small statistic values. The vertical gray lines mark deciles of the distribution for all features, so that if 10% of the features must be left out, then they are the ones with value of the filter statistic to the left of the first vertical gray line. The last vertical line leaves 1% of the data.

This can also be seen in figure 2, where the empirical distribution of the filter statistics grouped by the underlying hypothesis is displayed. The gray vertical lines indicate deciles of the filter, increasing from left to right, so that if 50% of the data is to be left out then all features with filter statistic up to the fifth gray line from the left are neglected. Thus, filter statistics that have the least overlap between their distributions under the null (blue line) and alternative (red line) hypotheses are expected to improve power, which is the case with the fold change filter. However, the opposite is true for the variance and signal filters, implying that these filter statistics tend to leave many non-null features out of the dataset. This is natural, as the fold change filter makes use of the group labels, which the other ones do not.

#### Filtering and fraction removed

An ideal filter only removes null features, thus decreasing the chance of making false positives. As a consequence the proportion of true null hypotheses, *π*_0_, decreases when compared to the whole set of features. So it is interesting to compare the filter statistics based upon the behaviour of *π*_0_(1- *γ*), as the proportion of features retained *γ *varies from 1 to 0.

**Figure 3:**
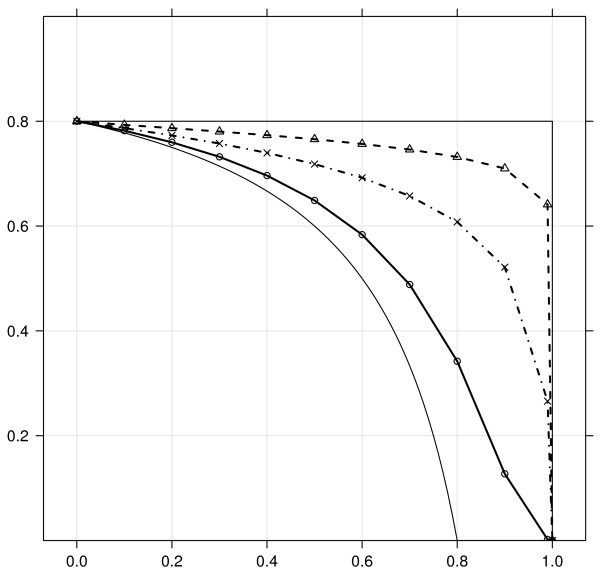
**Proportion of non-differentially expressed genes as function of the fraction filtered out *x *≡ 1 - *γ***. Each curve represents the mean of *π*_0_(*x*) over 1000 simulated datasets (error bars are small but not displayed for clarity). From bottom to top, curves represent the following situations: best filter (thin solid line), fold change filter (solid line, ○), signal filter (dashed-and-dotted line, ×), variance filter (dashed line, Δ) and the random filter (thin solid line).

As references, we consider both the best filter possible, which leaves out null features until there are none left, and a random filter, which leaves out null and non-null features with equal probability (see section "Simulation study setup" in Additional File 1 for detailed descriptions). In figure 3 *π*_0_(1 - *γ*) for the best and random filters serve as bounds below and above for all others filters. Amongst realistic filters, the best performances are obtained with the fold change filter, although it does not perform as well as the best filter. The variance filter performs worse than those, which is not surprising as it leaves proportionately more features out with small p-values than the others (figure 1). The worst performance is yielded by the signal filter, its performance slightly better than the random filter.

#### FDR and power

Since some filters are more likely to leave out alternative features than others, they will also have a different effect on power and achieved FDR. We evaluated the mean of each of these quantities across all 1000 simulated datasets.

**Figure 4:**
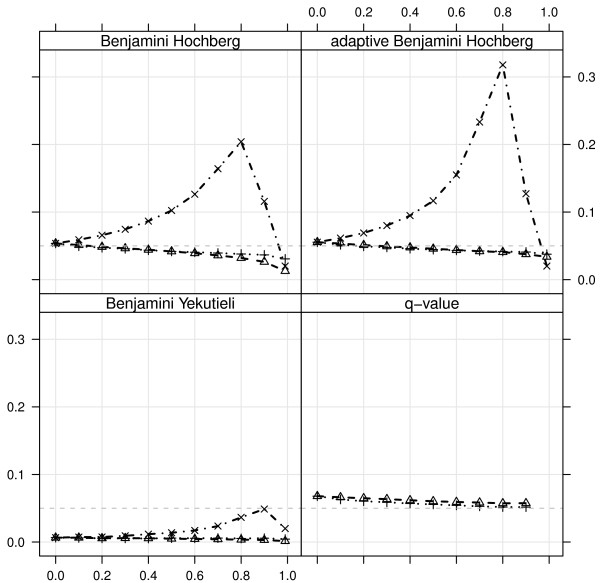
**Achieved FDR as function of the fraction filtered out *x *for the different filter statistics, fixing the FDR with each method at 0.05**. Values shown are the mean FDR over 1000 simulated datasets (the variability of the FDR is small - not shown). Filters are: dashed line = variance, dotted = signal, dashed-and-dotted = fold change. In all cases the proportion of non-differentially expressed genes is fixed at 0.8. The q-value method cannot be computed for the fold-change filter as in this cases the p-value range changes.

As the FDR is controlled at 5% in all cases, the achieved FDR is expected to remain constant as the proportion of filtered out features increases, but this is not always observed (figure 4). Indeed, when using either BH or aBH, the fold change filter yields an increase on the achieved FDR that gets larger as more features are filtered out. Interestingly, the variance and signal filters do not have this effect on the achieved FDR. A decrease on achieved FDR, while not as bad a problem as an increase since it means more conservative results, is also undesirable and is observed in some degree with all FDR methods. It is also noteworthy that two of the FDR methods (aBH and, to a smaller extent, qv) overestimate it even with no filtering, while one (BY) underestimates it over the entire range. The variance and signal filters showed the smallest induced bias on the FDR, for all methods considered. From the viewpoints of both bias at no filtering (*x *= 0) and trend for increasing values of *x*, using BH with variance or signal filter yielded the best results: the achieved FDR was closest to the required 5% for most filtered-out proportions *x*.

Our intuition also suggests that power should increase after filtering. Again here this is proven to not always hold (Additional File 1, figure S2). For example, the power yielded after using the signal filter almost invariably decreases, so that it is worse to use this filter than to not filter at all. The power yielded after using the variance filter increases slightly compared with no filtering, but the amount depends on the FDR method used. The fold change filter seems here to be the best, yielding considerable increases of power when used.

**Figure 5:**
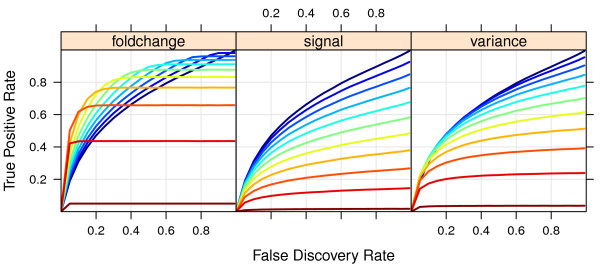
**Actual power (y-axis) displayed as function of the Benjamini-Hochberg FDR fixed at various levels, for the different filter statistics**. In each panel, one curve is displayed for each given fraction of features left out, varying from 0 (dark blue) to 0.9 (dark red) by steps of 0.1. In all cases the proportion of differentially expressed genes is fixed at 0.20. Note that all filters leave out some alternative features, so the maximum power achievable may be below 1 after filtering.

By considering both measures together a better picture emerges of the cost-benefit relationship of using each filter statistic. We construct an equivalent to a ROC curve for this (figure 5) using BH. To start with, the signal and variance filters always yield less power after any amount of filtering, a trend that gets stronger as the FDR threshold increases. On the other hand, the test statistic and fold change filters yield an improved power for each FDR thresh-old after filtering, for commonly used FDR threshold values up to 0.1 and filtered out ratios not larger than 0.5. Interestingly, the cost-benefit relationship between observed power and achieved FDR is constant for the fold change filter, regardless of the fraction filtered out *x*, whilst it deteriorates for the other filters as *x *increases (Additional File 1, figure S3).

#### Testing for filtering-induced FDR bias

**Figure 6:**
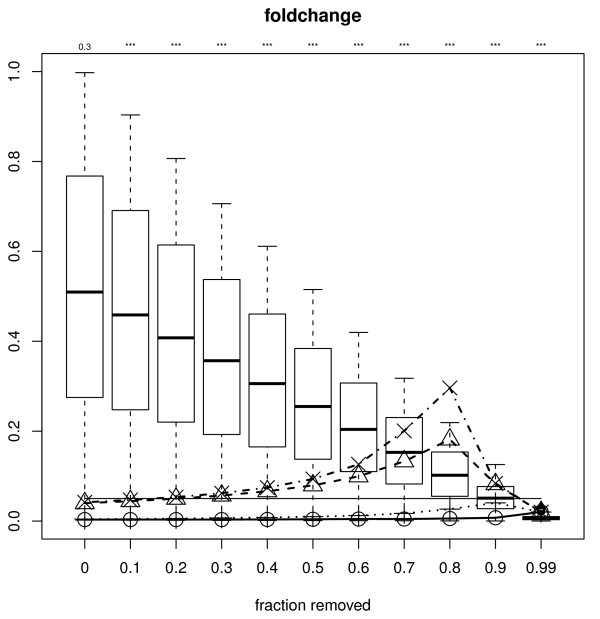
**Boxplots generated by null p-values yielded after permutation is applied to the simulated data, for varying proportions of features left in the data (x-axis) using the fold-change filter**. For comparison, the distribution yielded without filtering is shown (leftmost boxplot). Lines represent the achieved FDR using each of the methods aimed at 5% control level: BH (dashed line with triangles), aBH (dashed-and-dotted line with crosses), BY (dotted line). For comparison, the Bonferroni correction is also shown (solid line). Above each boxplot the p-value yielded by our test for FDR bias is given ('***' for < 0.001). The solid thin straight line at 5% represents the FDR threshold used.

The test for filtering-induced bias proposed (see Methods) can be easily applied to one of the simulated datasets. All filter statistics considered here are used, with a range of filtered out fractions (figure 6 and Additional File 1, figure S4). After permutation and filtering, signal and variance filters yield null p-values approximately uniformly distributed for all filtered-out fractions, whilst the fold-change filter does not, even if only 10% of the features are filtered out. Correspondingly, the FDR bias (computed on the data with no permutation) is larger for the two latter and negligible for the two former filter statistics, relatively for each FDR method. Note that the achieved FDR curves are very similar to those shown in figure 4, as expected. In a similar way, the corresponding observed power (not shown) follows the same curves as the ones shown in figure S2 of Additional File 1. With the p-values from the FDR bias test given, we can see that the situations where bias is introduced are correctly picked up.

Based upon these results, we conclude that the signal and variance filters introduce no significant FDR bias, but also yield little power gain. The fold change filter, on the other hand, does introduce bias on the FDR, but may yield improved power for *ϕ *< 0.1 and 1 - *γ *< 0.5. So, no filter statistic displays superior results all-round.

### Experimental data: childhood leukemia

In contrast to a simulation study, in this case it is not known which features are null or alternative. Since these are essential to measure achieved power and false discovery rates, we take the same approach as that from van Wieringen and van de Wiel [10], which is to choose an experiment with plenty of samples in each group to be compared, from which small subsets are selected and analysed. The idea here is to use the dataset with all available samples as the truth, so that the achieved power is estimated as the number of features found in the subset as well as in the whole data, divided by the total number of features found in the whole data. Similarly, the achieved FDR is estimated as the proportion of features selected in the subset that was not selected in the whole dataset.

We use a leukemia gene expression dataset described and first analysed by Den Boer *et al. *[11]. Briefly, the dataset consists of peripheral blood samples, from which RNA was isolated and hybridized to Affymetrix U133A microarrays, according to the manufacturers' protocol, and the data was subsequently pre-processed as described by Den Boer *et al. *[11]. We shall use the data corresponding to samples with the Tel-AML translocation (*n *= 44) and Hyperdiploid (*n *= 44) of their training cohort (*n *= 190). To compare the groups, we apply an empirical-Bayes linear regression model as implemented in the BioConductor package limma[12], and the yielded p-values are corrected for multiple testing by Benjamini-Hochberg's FDR [1]. We evaluate power and FDR bias in three study sizes (8, 16 or 24 samples selected at random per group) to check if sample size may affect results.

**Figure 7:**
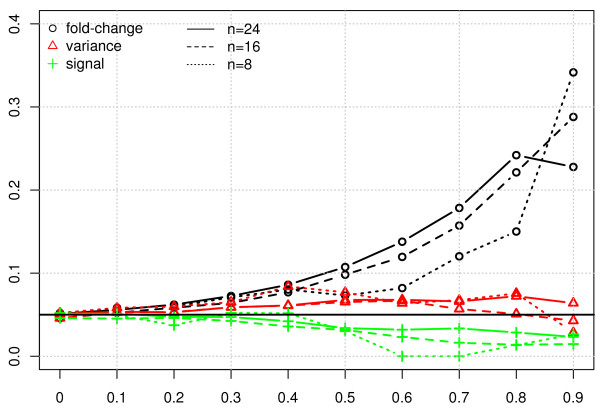
**Achieved FDR as function of the fraction filtered out for the different filter statistics, using an FDR control level fixed at 0.05 (horizontal solid, black line)**. Computations are done using randomly selected subsets of *n *= 8, 16, 24 samples from each subtype considered. Differential expression is evaluated using limma, and p-values are FDR-corrected.

The true positive rate and the achieved FDR were calculated for various filter thresholds ranging from 0-0.9 using the fold-change, variance and signal filters (see figure 7 and Additional File 1, figure S5). With the FDR level fixed at 5%, as the fraction filtered out increased the achieved FDRs with both the signal and variance filters remained stable around 5%, but increased with the fold change filter, in agreement with the simulation study results (upper-left panel in figure 4). Interestingly, this FDR bias seemed invariant to the sample size. On the other hand, there is a strong relationship between sample size and observed power (true positive rate), in spite of the model being used taking advantage of the large number of genes in the study to improve power for detection of differential expression. For each fixed sample size, however, results are similar to those for the simulation study, with the fold change filter improving the power but the signal and the variance filter having no or the opposite effect. These confirm our conclusions that power increase via filtering is linked to an FDR bias.

## Results and Discussion

Filtering features is a common practice in high-dimensional data analysis, aimed at minimizing the penalty due to multiple testing correction and, consequently, increasing power. As we have shown in this paper, an increase in power is linked to introducing bias on the FDR. This is because any filter statistic that filters out only noise is bound to be associated to the test statistic and, therefore, affects the null distribution of p-values and introduces FDR bias.

The fact that filtering introduces FDR bias is evident from published articles. For example, Querec and co-authors [13] used a fold-change filter to increase their list of 22 differentially expressed genes to 65, both obtained with 5% FDR. Of the longer list 33 genes are validated by RT-PCR, of which 26 are confirmed, yielding in fact an FDR between 11 and 20%. The sought power increase could have been achieved instead by using a more suitable analysis model for their data than a per-gene ANOVA, such as the one proposed by Smyth [12] and implemented in the BioConductor package limma.

We have assumed in this study that a Student's t test, or an empirical Bayes linear regression model, is used to find differential gene expression between the two groups. However, results are not specific to these models. Indeed, had the Wilcoxon rank-sums test been used similar results would be produced. To illustrate this, we show power and achieved FDR computed in our simulation study using this test statistic and the Benjamini-Hochberg FDR (see Additional File 1, figure S6). The FDR curves (left pannel) and the power curves (right pannel) are very similar to the ones produced using the t-test statistic.

The conclusion that FDR bias may result from the use of filtering is also not dependent upon the shape of the alternative hypothesis. Indeed, in much the same way as for the two-sided alternative, for a one-sided alternative such as is the case when an F test is used in ANOVA, it still holds that whenever *G^W ^*≠ *G *the F-test p-values do not follow a uniform distribution under *H*_0_.

Filtering can also affect the fit of models that estimate the distribution of one (or more) parameters across all genes, such as empirical Bayes models like the one proposed by Smyth [12]. Indeed, such models rely on a large number of features with certain common characteristics, and if for example half of the features with small variance are left out, it can be that the distribution for the sample variance may no longer be well-described by the model.

Other authors have also attempted to handle the effect of filtering on multiple testing correction. McClintick *et al. *[14] used permutations to estimate the number of false positives, but ignored the fact that if a filter is used the null distribution of p-values may be affected. Hackstadt and Hess [15] also propose a framework that makes objective use of the p-value distribution, but assumed without criticism that power is increased after filtering. Our proposed framework allows us to not only demonstrate that an FDR bias may be introduced by filtering, yielding important understanding about the problem, but also to evaluate this bias, and its effect on power, using a variety of FDR formulations and filter statistics.

To the best of our knowledge, we are the first to propose a statistical test to check if filtering introduces FDR bias. It can be used in any application for any combination of statistical model, filtering setup and FDR method. In our simulation study filter statistics that use group information are found to introduce bias, whilst those approximately independent from group information do not, as expected. We suggest researchers use this test to make decisions of whether or not to apply any filtering to the data.

Our FDR bias test differs from examining the density of the left-out values as mentioned elsewhere [15]. We believe that, as these p-values typically include a (hopefully, but not always, small) set corresponding to non-null features, even if the filtered-out p-values do have an empirical distribution close to the uniform, it can still be that the FDR is biased.

A violation of the uniform null distribution assumption also occurs when there is correlation among features, as previously pointed out by [16,17]. This served as motivation to propose resampling-based FDR procedures which preserve the original dependence structure among features. We checked if these FDR-estimating methods would be affected by filtering in our simulation study (see Additional File 1, section 12 and figure S7 therein). Our conclusion is that the methods tested do not avoid introducing FDR-bias as a result of filtering in the context considered. Further research would be needed to better understand the behaviour of these FDR methods when filters are used.

On the basis of our results, we believe it is unlikely that a two-step approach involving testing and filtering improves power and does not bias the FDR. Our conclusion is thus that two-step approaches should be avoided in general, extending to a general microarray study the conclusions of Pounds and Cheng [18] that "the use of even the best filter may hinder, rather than enhance, the ability to discover interesting probe sets or genes", obtained for filters such as present/absent calls (Affymetrix microarrays) using simulation and experimental data.

It often occurs that researchers wish to prioritize features via fold change, say, from a compiled list of differentially expressed genes, estimated to contain a fixed percentage *ϕ *false positives, with the goal of making a shorter list for in-lab validation. While this does not bias the multiple testing correction as is done a *posteriori*, researchers should be aware that the shorter list is no longer expected to have the same percentage *ϕ *of false positives. Here we note that, in our simulation study, for some combinations of FDR estimation method and filter the FDR was preserved after post-FDR filtering, but no power improvement resulted (see Additional File 1, section 13 and figures S8 and S9 therein). A better alternative would be to incorporate the fold-change filter threshold into the statistical model used, as suggested by McCarthy and Smyth [2] and Zhang and Cao [3]. A similar approach could be used to derive a statistical test that combines a two- or multiple-group comparison and the variance filter, based upon the F statistic. In general, however, for each choice of statistical model and filter statistic a new combined model needs to be worked out.

Alternatives, for any generic filter and test, to avoid filtering-induced FDR-bias would be to adapt the multiple testing correction method to relax the assumption of uniform distribution for the null features in a way that filtering-induced bias is avoided, or to devise a way of correcting the FDR bias. These issues deserve further research if two-step approaches are to yield correct results.

## Conclusion

We showed both in theory and in applications that, when a statistical test follows a filter to prioritize features for further analysis, power increase is linked to an FDR bias, making results look too optimistic. Our proposed statistical test for FDR bias can be used to guide researchers in their decision as to whether or not to filter, and as to the filter setup to use, such as the filter statistic and the proportion of features filtered out.

## Software

All the computations were performed using R version 2.10.0 [19] and the BioConductor (2.5) packages multtest (2.2.0), qvalue (1.19.1) and genefilter (1.28.0). All the figures were made using basic R graphics and packages geneplotter (1.24.0), lattice (0.17-26) and RColorBrewer (1.0-2). All scripts used here are available from the authors upon request. R scripts and functions implementing the simulation and reproducing the figures and results presented here can be found at: http://www.humgen.nl/MicroarrayAnalysisGroup.html.

## Authors' contributions

MvI has developed the framework and performed all computations. JMB participated in discussions and coordination. RXM gave the original idea and supervised the work. All authors collaborated in writing, read and approved the final manuscript.

## Supplementary Material

Additional file 1 **This document contains details of some of the theoretical developments in the article, as well as figures**. Click here for file
