# Supplementary material for: Filtering, FDR and power
Source: BMC Bioinformatics. 2010 Sep 7;11:450. doi: 10.1186/1471-2105-11-450 (PMC2949886; doi:10.1186/1471-2105-11-450)
Supplement: Additional file 1 — This document contains details of some of the theoretical developments in the article, as well as figures. [file 1471-2105-11-450-S1.PDF]

Filtering, FDR and power: Additional File 1

M. van Iterson , J.M. Boer , R.X. Menezes*

Email: R.X. Menezes*- r.menezes@vumc.nl;

*Corresponding author

1 Study design

Here we assume a study setup commonly found in practice, involving gene expression profiles of two groups of independent samples,with the null hypothesis representing no differential expression between the two groups for any given gene, and a corresponding two-sided alternative. With notation, assume that the two independent groups of samples have sample sizes n_X, n_Y ($n_Z = n_X + n_Y$) and that each sample has a corresponding mRNA profile including m features (probes or genes, say). So the data consists of two matrices, $X = [X_1, X_2, \ldots, X_{n_X}]$ and $Y = [Y_1, Y_2, \ldots, Y_{n_Y}]$. Let $Z = [X, Y]$ be the matrix including all samples from both groups. Gene-wise sample means are represented by $m \times 1$ vectors $\bar{X}_{n_X}, \bar{Y}_{n_Y}$ for each group separately, and by $\bar{Z}_{n_Z}$ for all samples. Similarly, gene-wise sample variances are represented by $m \times 1$ vectors S^2_X, S^2_Y for each group separately and by S^2_Z for all samples.

A test statistic $M = M(Z)$ is computed to test the null hypothesis $H_0 : \mu_X = \mu_Y$ against the alternative $H_a : \mu_X \neq \mu_Y$. If V is a binary $m \times 1$ vector with $V_i = 1$ whenever H_0 holds for feature i, and $V_i = 0$ otherwise, then $\pi_0 = (1^t V)/m$ represents the proportion of features that follow H_0. Then the distribution $\mathcal{F}$ of M can be written as

$$M \sim \pi_0 \mathcal{F}_0 + (1 - \pi_0)\, \mathcal{F}_a, \tag{1}$$

where $\mathcal{F}_0, \mathcal{F}_a$ are evaluated under H_0, H_a, respectively. We shall represent by f and F the probability density function (pdf) and the cumulative density function (cdf) corresponding to $\mathcal{F}$, respectively where, as above, indices when used indicate the holding hypothesis. Here we assume that $\mathcal{F}_0$ is a symmetric distribution around a value k for convenience, i.e., its density function satisfies $f(k - u) = f(k + u)$ for a given k and all u, both real numbers.

Let also $P = (P_1, P_2, \ldots, P_m)^t$ be the vector of p-values computed for the observed data. Then we can

write that the distribution $\mathcal{G}$ of P is a mixture between its distribution under H_0 and under H_a, respectively $\mathcal{G}_0, \mathcal{G}_a$, i.e.,

$$P \sim \pi_0 \mathcal{G}_0 + (1 - \pi_0)\mathcal{G}_a, \tag{2}$$

and it is a well-known fact that $\mathcal{G}_0 = \mathcal{U}[0, 1]$. Similarly to the test statistic, G, g shall represent the cdf and pdf of $\mathcal{G}$ respectively.

2 Selecting features - the filter

Features may be selected from the data to be kept in the analysis. Such selection is defined by a filter statistic $W = W(Z)$, a measure to be computed from the data, and a threshold w that indicates if the feature is kept or not. Here we are mostly concerned with evaluating the effects of different types of filter statistics, so we shall keep filter thresholds w fixed and, whenever possible, comparable across various filter statistics. In formulae below subscripts will be omitted when they are obvious, for the sake of clarity. For given choices of W, w, applying the filter to the dataset is equivalent to evaluating the indicator function $I\{W(Z_i) \geq w\} \equiv R_i$ for each feature i, yielding $R = (R_1, R_2, \ldots, R_m)^t$, a $m \times 1$ vector indicating which features are left in the data. Thus the fraction of the total number of features taken further for analysis is $\gamma = (1^t R)/m$.

In this context, the filtered dataset and p-values can be represented by $Z^W = \text{diag}\{R\}Z$ and $P^W = \text{diag}\{R\}P$, respectively, where $\text{diag}\{R\} \equiv \text{diag}\{R_1, R_2, \ldots, R_m\}$, a diagonal matrix of the elements of R. From (1), the distribution $\mathcal{F}^W$ of M^W, the test statistic after filtering, is given by the mixture

$$M^W \sim \pi_0^W \mathcal{F}_0^W + (1 - \pi_0^W)\mathcal{F}_a^W, \tag{3}$$

where $\pi_0^W = (1^t \text{diag}\{R\}V)/m$ represents the proportion of null features after filtering. Similarly for the filtered p-values, we have from (2)

$$P^W \sim \pi_0^W \mathcal{G}_0^W + \left(1 - \pi_0^W\right) \mathcal{G}_0^W. \tag{4}$$

3 Distribution of p-values

It is expected that, after filtering, the threshold for significance will increase. However, it does not necessarily follow that the filtered data yields more power to detect differential expression, as some filter statistics may leave out features that do not follow H_0. In addition, the increased significance threshold may no longer correctly estimate the error, if the filtering method does not select null p-values from the

entire range with equal probability as we have shown above. In order to evaluate these aspects, we need to consider the effect each filter statistic has on the p-values distribution, under both H_0 and H_a.

First let us consider how p-values are generated. Assume for simplicity that the analysis used involves a symmetric distribution around 0 $\mathcal{F}_0$ for the test statistic M and a two-sided H_0 rejection region. This is the case in most studies as the one we consider here. Then the p-values are given by

$$P = 2\left[1 - F_0\left(|M|\right)\right]. \tag{5}$$

In particular, for null features we have $P_0 = 2\left[1 - F_0\left(|M_0|\right)\right]$, where M_0 represents the test statistic M under H_0, so that it follows $\mathcal{F}_0$. It is well known that this implies that $P_0 \sim \mathcal{U}[0,1]$. One possible proof follows.

Let $M \sim \mathcal{F}$ with corresponding cdf F, and suppose we are testing $H_0 : E(M) = 0$ against $H_a : E(M) \neq 0$. Then a p-value after observing $M = x_0$ can be calculated as

$$\begin{aligned} P &= P\{M > |x_0|\} = P\{M > |x_0|\} + P\{M \leq -|x_0|\} \\ &= 1 - P\{M \leq |x_0|\} + P\{M \leq -|x_0|\} \\ &= 1 - F(|x_0|) + F(-|x_0|) \\ &= 1 - F(|x_0|) + 1 - F(|x_0|), \end{aligned}$$

where we have used the fact that $\mathcal{F}_0$ is symmetric around zero, meaning that $F(-u) = 1 - F(u)$.Thus we have, for any possible value of M, that the p-value is

$$P = 2[1 - F(|M|)].$$

The distribution of P can now be evaluated as follows:

$$\begin{aligned}
G_P(p) &= P\{2[1 - F(|M|)] \leq p\} \\
&= P\{1 - F(|M|) \leq p/2\} \\
&= P\{1 - p/2 \leq F(|M|)\} \\
&= P\{F^{-1}(1 - p/2) \leq |M|\} \\
&= 1 - P\{|M| \leq F^{-1}(1 - p/2)\} \\
&= 1 - \{F[F^{-1}(1 - p/2)] - F[-F^{-1}(1 - p/2)]\} \\
&= 1 - \{F[F^{-1}(1 - p/2)] - 1 + F[F^{-1}(1 - p/2)]\} \\
&= 1 - \{2F[F^{-1}(1 - p/2)] - 1\} \\
&= 2 - 2F[F^{-1}(1 - p/2)] \\
&= 2 - 2(1 - p/2) \\
&= p.
\end{aligned}$$

So, we can conclude that p-values have a uniform distribution over $[0, 1]$, so long as the distribution of the test statistic M is F, the same used to compute the p-values. Similar proofs can be obtained for the simpler cases of one-sided alternatives.

After filtering, a similar relation holds. Indeed, we would then have $P_0^W = 2\left[1 - F_0\left(|M_0^W|\right)\right]$, where M_0^W has cdf F_0^W. Then the cdf for P_0^W is

$$G_0^W(p) = 2\left\{1 - F_0^W\left[F_0^{-1}(1 - p/2)\right]\right\} \tag{6}$$

and, in case the filter selects null p-values from the entire range with equal probability, we have $F_0^W = F_0$ and $P_0^W \sim \mathcal{U}[0, 1]$, as before filtering. However, if $F_0^W \neq F_0$, the cdf of the p-values does not correspond to the uniform. How far it is from the uniform depends on how the filter statistic W used affects F_0. In order to evaluate this, we must consider a test statistic and the commonly used filter statistics given in the Methods section of the main text.

4 Filtering and p-values distribution

Once an expression for the pdf of the test statistics after filtering is obtained, we can obtain the pdf and cdf of the p-values using the relation $P = 2[1 - F_0(|M|)]$ (see section "Distribution of p-values" of this

document). For expressions corresponding to some of the filter statistics, see section "Density of test statistics after Filtering" in this document.

$$G_b^W(p) = 1 - F_b^W\left(F_0^{-1}(1 - p/2)\right) + F_b^W\left(-F_0^{-1}(1 - p/2)\right) \tag{7}$$

and

$$g_b^W(p) = \frac{1}{2}\frac{\left(f_b^W\left(F_0^{-1}(1 - p/2)\right) + f_b^W\left(-F_0^{-1}(1 - p/2)\right)\right)}{f_0\left(F_0^{-1}(1 - p/2)\right)}. \tag{8}$$

By plugging-in the derived pdf and cdf of the filter statistics, we can display the effect of each filter on the null and alternative distributions of p-values (see figure 1 of the main text).

5 FDR methods and p-values distributions

We have pointed out that filtering may alter the null distribution of p-values under certain conditions (see section 2.1 of the main text). So in order to evaluate the effect of filtering on the FDR, it is useful to express it in terms of the p-values cdf, which is possible in some cases [1]. Indeed, the BH, aBH and BY methods can all be re-written as follows: to control the FDR at ϕ, reject all hypotheses i in the set

$$\{i : P_i \leq u^*(\phi)\}, \tag{9}$$

where

$$u^*(\phi) = \max_u \{g(u, \phi) \leq G(u)\}, \tag{10}$$

G represents the general p-values cdf, and

$$\begin{aligned} g(u, \phi) &= u/\phi, \quad \text{for BH, and,} \\ g(u, \phi) &= (\pi_0)u/\phi, \quad \text{for aBH.} \end{aligned}$$

Note that, if all features are null, the set given by 9 is likely to be empty for all $g(u, \phi)$ considered.
An intuition behind this representation follows. If all features are null and independent, the p-values cdf G will correspond to that of a uniform in $[0, 1]$, represented by a straight line from $(0, 0)$ to $(1, 1)$. If these assumptions are violated, typically G will become concave with $G(u) >= u$ for $u \in [0, 1]$. The Benjamini-Hochberg FDR can be seen in this context as multiplying the angle of the null cdf by $1/\phi$, so that $G(u) \geq u/\phi$ is expected to involve by chance $\phi\%$ null features. Under the adaptive Benjamini-Hochberg, the angle is corrected by the proportion of null features, becoming less strict.

Benjamini-Yekutieli's method can be expressed in a similar form, with $u_i^*(\phi)$ instead of $u^*(\phi)$ in (9), where

$$u_i^*(\phi) = \max_u \{g(u_i, \phi) \leq G(u)\} = \max_u \left\{ \frac{u}{\phi} \sum_{j=1}^{i} \frac{1}{j} \leq G(u) \right\},$$

which means that, in this case, the angle correction depends on the cardinality of the set of selected p-values.

Here $g(u_i, \phi)$ represents the secant establishing which part of the cdf G_m can be generated by chance, and which part cannot. The functional form of the function gives some insight into how each FDR method works: for BH the threshold depends on ϕ only, for aBH it also involves the proportion of null features and, finally, for BY it is a function of how many features are selected.

From section 3 of this supplement we have that the cdf for p-values is given by

$$G(p) = 2\left\{1 - F_0\left[F^{-1}\left(1 - p/2\right)\right]\right\}, \quad 0 \leq p \leq 1, \tag{11}$$

where F, F_0 represent the general and the null cdf's of the test statistic. Replacing 11 into 10 yields

$$u^*(\phi) = \max_u \left\{F_0^{-1}\left[1 - g(u, \phi)/2\right] \leq F^{-1}(1 - u/2)\right\},$$

which can be evaluated for given F, F_0.

If we define, for each hypothesis $i (i = 1, \ldots, m)$,

$$I_i = \begin{cases} 1 & \text{if } \; P_i \leq u^*(\phi) \\ 0 & \text{if } \; P_i > u^*(\phi) \end{cases}$$

then the expected number of rejected hypotheses, which is the expected cardinality of the set 9, can be evaluated as

$$E\left(\sum_{i=1}^{m} I_i\right) = \sum_{i=1}^{m} E\left(I_i\right) = \sum_{i=1}^{m} P\left\{P_i \leq u^*(\phi)\right\}. \tag{12}$$

The above development can also be used in the general case where alternative features are observed.

6 FDR and Power as function of fraction filtered out

The total number of hypotheses is m of which m_0 are calculated under H_0 and m_1 under H_1. If we assume that the total number of hypotheses will decrease linearly with the fraction $1 - \gamma \equiv x$ filtered out we get for $m(1 - \gamma)$:

$$m(1 - \gamma) = m\gamma \quad 0 \leq \gamma \leq 1 \tag{13}$$

Now we assume an ideal filter removing first only features that follow H_0. When $\gamma = 1 - \pi_0$, all null features are removed and

$$m_0(x) = \begin{cases} m_0 - mx & 0 \le x \le \pi_0 \\ 0 & \pi_0 < x \le 1 \end{cases}$$

$$m_1(x) = \begin{cases} m_1 & 0 \le x \le \pi_0 \\ m(1-x) & \pi_0 < x \le 1 \end{cases}$$

The proportion of features under H_0 as a function of the fraction removed is given by:

$$\pi_0(x) = \begin{cases} \frac{\pi_0 - x}{1-x} & 0 \le x \le \pi_0 \\ 0 & \pi_0 < x \le 1 \end{cases}$$

The original FDR correction method of Benjamini-Hochberg can be re-written as an inequality involving the mixture distribution of p-values, $G_m(u)$:

$$u^* = \max\left\{ {}^u/_\phi \le G_m(u), 0 \le u \le 1 \right\}, \tag{14}$$

where u represents possible values of the random variables $\{P_i\}$

The mixture distribution consists of a uniform distribution and an alternative distribution with mixture coefficient π_0.

$$G_m(u) = \pi_0 u + (1 - \pi_0) G_a(u)$$

By plugging in a suitable functional form for the alternative distribution it is possible to solve (14) for u^*. Langaas and others suggest a beta-distribution for the alternative $Beta(1, b)$. The choice of $a = 1$ makes (14) difficult to solve. But when $b = 1$ and $0 < a < 1$ the equation is easily solved and the alternative distribution still seems realistic. First plugging in the proportion of hypotheses under H_0 as function of the fraction removed, $\pi_0(x)$:

$$u^* = \max\left\{ {}^u/_\phi \le \frac{\pi_0 - x}{1-x} u + \frac{1-\pi_0}{1-x} u^a \right\} \tag{15}$$

We immediately see the $u^* = 0$ is the trivial solution, but there also exists a solution $0 < u^* < 1$, which is given by

$$u^* = \left(\frac{1-x}{\phi(1-\pi_0)} - \frac{\pi_0 - x}{1-\pi_0} \right)^{1/(a-1)} \tag{16}$$

For (16) to hold we must assume that the filter applied is an ideal filter removing null hypotheses only first. And when we are left with only alternative hypotheses the FDR remains constant.

7 Density of test statistics after filtering

Let $Z = g(X, Y)$ be a function of X and Y, two other independent random variables. If the marginal distribution functions of X, Y are known, then the follow formula can be used to derived the probability density of Z:

$$f_Z(z) = \int \frac{\partial g^{-1}(z;y)}{\partial z} f_X(g^{-1}(z;y)) f_Y(y) dy \tag{17}$$

For example: Student t, $T = \frac{Z}{\sqrt{V/n}} = g(Z, V) \Leftrightarrow g^{-1}(T;V) = T\sqrt{V/n} = Z$ and using $\frac{\partial g^{-1}(T;V)}{\partial t} = \sqrt{v/n}$ we get:

$$f_T(t) = \int_0^\infty \sqrt{v/n} \frac{e^{-1/2(t\sqrt{v/n})^2}}{\sqrt{2\pi}} \frac{v^{\frac{n}{2}-1} e^{-n/2}}{\Gamma\left(\frac{n}{2}\right) 2^{\frac{n}{2}}} dv = \frac{\Gamma\left(\frac{n+1}{2}\right)}{\Gamma\left(\frac{n}{2}\right)\sqrt{n\pi}} \left(1 + t^2/n\right)^{1/2(n+1)}, \tag{18}$$

where the integration limits are over the support of V.

If either X, Y or any transformations of these are the filter statistics, we can derive in a similar fashion as equation (18) the distribution of the test statistics after filtering. For example if the filter statistics is X and, say, we filter on the absolute value of $|x|$ the integration will be over the support of Y excluding a region according to $I(|x| \geq \omega) = I\left(|g^{-1}(z;y)| \geq \omega\right) = I(|y| \geq h(z, \omega))$:

$$f_Z(z) = \int I(|y| \geq h(z, \omega)) \frac{\partial g^{-1}(z;y)}{\partial z} f_X(g^{-1}(z;y)) f_Y(y) dy \tag{19}$$

In case of the Student's t test statistics, filtering on the absolute value of the fold-change results in:

$$f_T(t) = \int_0^\infty I\left(|t\sqrt{v/n}| \geq \omega\right) \sqrt{v/n} \frac{e^{-1/2(t\sqrt{v/n})^2}}{\sqrt{2\pi}} \frac{v^{\frac{n}{2}-1} e^{-n/2}}{\Gamma\left(\frac{n}{2}\right) 2^{\frac{n}{2}}} dv \tag{20}$$

$$f_T(t) = \int_{\frac{n\omega^2}{t^2}}^\infty \sqrt{v/n} \frac{e^{-1/2(t\sqrt{v/n})^2}}{\sqrt{2\pi}} \frac{v^{\frac{n}{2}-1} e^{-n/2}}{\Gamma\left(\frac{n}{2}\right) 2^{\frac{n}{2}}} dv \tag{21}$$

The last integral can be re-written as the product of a central Student's t density and a modified gamma-distribution function $f_T(t;n) F_\Gamma(1 + n/t^2; 2/\omega^2, n+1/2)$. The density function for other filters that have a relation to the test statistics are derived similarly. Under the alternative the derivations becomes more difficult and numerical integration is used for evaluation of the density function after filtering.

8 Simulation study setup

To investigate the properties of the filtering on high-dimensional datasets, a simulation study is carried out. We use a setup that mimics a microarray experiment where thousands of genes are measured simultaneously, based upon a setup first suggested by [2].

A total of $m = 5000$ features were generated independently for each of $n = 10$ samples per group. Let R_{ij} and S_{ij} , $i = 1, \ldots, m$, $j = 1, \ldots, n$ be random variables $S_{ij} \sim N\left(-\mu_i/2, 1\right)$ and $R_{ij} \sim N\left(\mu_i/2, 1\right)$ with the first $\mu_i = 0$ for $i = 1, \ldots, m_0$ and the remaining μ_i for $i = m_0 + 1, \ldots, m$ were drawn from a symmetric bitriangular distribution as described by [2]. A total of $N = 1000$ datasets were simulated with the proportion of non-differentially expressed features fixed at $\pi_0 = 0.8$. Additional noise is added to the model using $X \sim \log(e^R + e^\epsilon)$ and $Y \sim \log(e^S + e^\epsilon)$ with $\epsilon \sim N(0, 0.01)$, so that the noise is positive and additive. Two-sample Student-t test statistics were calculated and converted to two-sided p-values accordingly.
FDR-based multiple testing correction methods used were the ones suggested by: Benjamini-Hochberg [3], Benjamini-Yekutieli [4], adaptive Benjamini-Hochberg [5] and Storey [6]. For each simulated dataset, both the achieved FDR (fraction of false positives amongst features below FDR threshold) and the observed power (fraction of p-values below FDR threshold belonging to non-null features, out of all 4000 non-null features) were calculated.
Dedicated R [7] functions were used in the computations. Specifically, for the multiple testing correction methods the `multtest` and `qvalue` package were used.
In order to compare the different filter methods the same fraction was removed based on the empirical quantiles of the filter statistics. Note that FDR estimation using the qvalue method relies on p-values taking values on the entire $[0, 1]$ interval. Thus, we only used the variance and signal filter statistics with this method. Using the bootstrap method for estimation of the optimal λ [6] does not the make the FDR estimable.

8.1 Illustration of filter statistics

We shall show how each filter statistic affects power and FDR estimates using one of the simulated datasets chosen at random.
In figure 1 we can see that the filter statistics used have little association with each other, as evidenced by the lack of pattern described by the dot clouds. This implies that they indeed look at different aspects of gene expression. It is also clear that, when using any of these filter statistics, there is always a fraction of the differentially expressed features that is left out, for which the power is automatically set at zero.

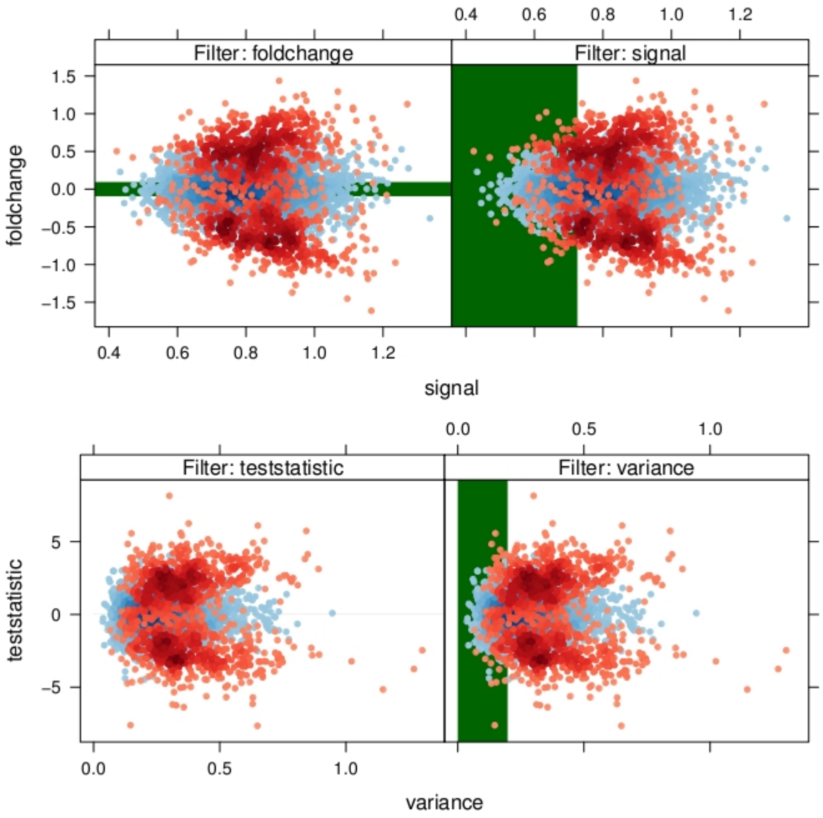

Figure 1: Relationship between filter statistics, with each dot representing one feature and its colour indicating the hypothesis: blue = H_0, red= H_a. The scatterplot on the left panel is the same as the one on its right. The green area underlying the plots marks the features that would be filtered out if 25% of them were to be excluded, using each filter. The filter in the bottom-left hand corner uses the test statistic itself, and is included to yield a contrast with the variance filter.

8.2 Relationship between achieved FDR and power

Here we examine further aspects of power and observed FDR variation, as a result of increasing proportions of features being left out of the data. Firstly, we see from figure 2 that the power displays similar behaviour to that of the FDR (figure 5 in main text), as expected. Indeed, FDR methods yielding larger observed FDR do so because they involve larger thresholds for significance, which tend to yield also a larger power. What we can also see is that this depends on the filter statistic, as for the FDR: the ones that introduce the least bias on the FDR also yield the lowest power.

Another important aspect to examine is the relationship between observed power and FDR, for different filter statistics (3). Here we use only BH for FDR correction for illustration, with results obtained using other methods yielding similar conclusions. Filter statistics that are more closely related to the hypothesis

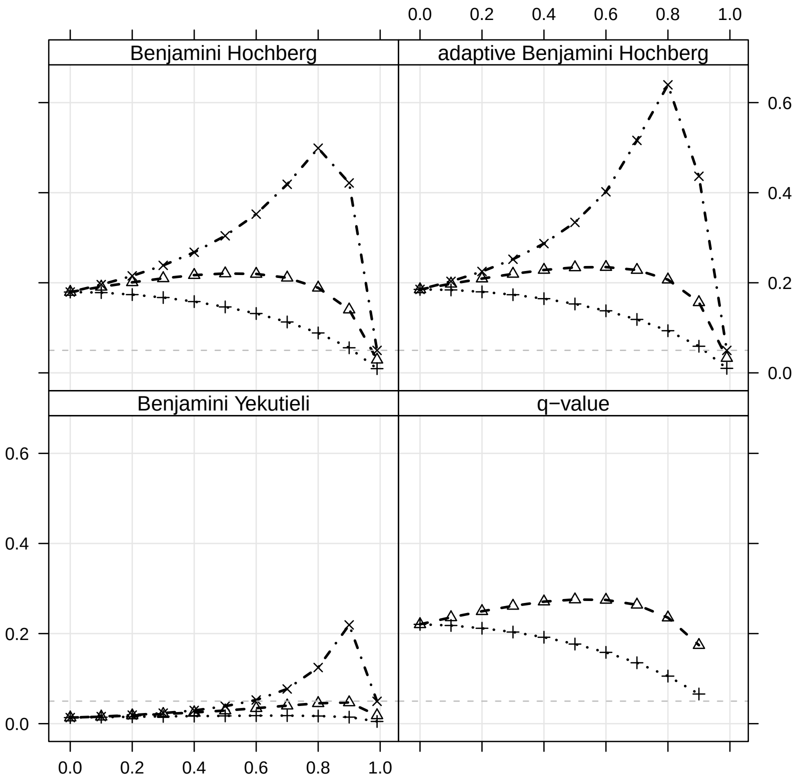

Figure 2: Actual power as function of the fraction filtered out for the different filter statistics, fixing the FDR with each method at 0.05. Values shown are the mean observed power over 1000 simulated datasets (the variability of the power is small - not shown). Filters are: solid line = fold change, dashed = signal, dashed-and-dotted = variance, dotted = t-test statistic. This last filter is included for comparison with the fold-change filter. In all cases the proportion of differentially expressed genes is fixed at 0.20. Results of the q-value method could only be obtained for the variance and signal filter, as for the fold change and test-statistic ones the p-value range was reduced, violating an assumption of the method.

tested (left hand-side panels in the figure) yield essentially the same relationship between achieved power and FDR, regardless of the filtered-out proportion. This means that the cost-benefit relation of using filtering remains the same. On the other hand, for the filter statistics that are unrelated to the hypothesis tested (right hand-side panels) the cost-benefit-relationship gets worse, with the power decreasing for increasing proportions of left-out features.

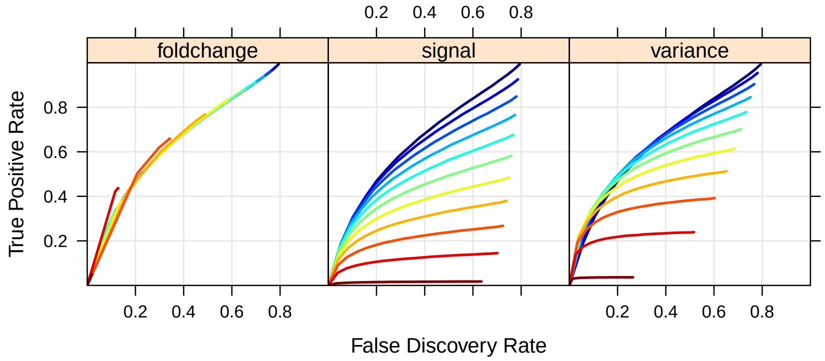

Figure 3: Relationship between actual power (y-axis) and achieved FDR(x-axis) for various filtered-out fractions (0, black; then in gray shades increasing by steps of 0.1) for the different filter statistics, fixing the FDR control with the BH method at 0.05. In all cases the proportion of differentially expressed genes is fixed at 0.20.

9 A test for filtering-induced FDR bias

9.1 The test

In section 2.1 of the main text, we have shown that a necessary condition for the null distribution of p-values after filtering to still follow a $\mathcal{U}[0,1]$ is that the filter selects null features at random from their entire range $[0,1]$. This motivates a hypothesis test to check in practice if this condition holds.

Let $P_0 \sim \mathcal{U}[0,1]$ be the $m \times 1$ vector of p-values generated if all features in the data followed H_0. Then generate a number N_0 of observations P_{0i} of this vector by permuting row and column labels of the data. For each permutation, apply the filter W to the data, leaving only γm of the entries in each P_{0i}. Subsequently, compare each shortened vector P_{0i} to its null distribution $\mathcal{U}[0,1]$ by means of a Kolmogorov-Smirnov test, generating p-values $(Q_1, Q_2, \ldots, Q_{N_0}) \equiv Q$. If the filter selects null features randomly from their entire range, then it follows that $Q \sim \mathcal{U}[0,1]$, which can again be assessed by a Kolmogorov-Smirnov test.

In order to enable fair estimation of the null p-values distribution, we permute column labels for each row, independently per row. Here the column label permutation eliminates any association between gene expression levels and clinical variables, whilst applying the permutations independently per row eliminates associations between probes.

Another important point is to find filter effects on the null p-value distribution that yield under-estimation of the FDR. This is considered to be the worst error, making results look more optimist than they really are. In order to do so, we compare the empirical distribution of the filtered null p-values $\hat{G}_0^W$ to the

uniform $\mathcal{U}$, against the alternative that the former is super-stochastic, i.e. $\hat{G}_0^W(u) \geq G_0(u)$ for all $u \in [0, 1]$. This can be done with the Kolmogorov-Smirnov test, as Benjamini-Hochberg's FDR step-up method [3] is equivalent to it with this alternative hypothesis [8]. Our implementation uses this step. Obviously, it is also possible to test for effects in both directions.

9.2 Illustration with simulation

The performance of the test can be illustrated by simulation in the following way. Let us consider one dataset from our simulation study, with by construction 20% differentially expressed probes. We then filter varying proportions of probes out of the dataset, using the various filter statistics. In each case, we evaluate the achieved FDR.

We next permute column labels independently per probe, generating a dataset with no differential expression. Now varying ratios of probes are again filtered out from the data, and the empirical distribution of the (null) p-values is compared to the uniform by a Kolmogorov-Smirnov test. This is repeated for 1000 permutations, yielding a distribution of the Kolmogorov-Smirnov p-values.

As can be seen from figure 4, our test tends to not reject the null hypothesis that the p-values follow a uniform distribution, when signal or variance filters are used. Coherently, in these cases the achieved FDR remains approximately constant even after filtering. On the other hand, with the fold change filter the FDR-bias test indicates clearly that a potential bias can be introduced, which is confirmed by the achieved FDR increasing with the filtered-out fraction, whilst the FDR control level was kept fixed.

10 Experimental data: childhood leukemia

Here we show two additional figures obtained with this dataset. First in figure 5 we display the estimated power achieved in the analysis, as a function of the fraction of features filtered out of the data. We use the same filter statistics as before, namely the fold change, the overall variance and the signal. We consider three subsets of randomly selected samples from each subtype, of sizes 8, 16 and 24 respectively. In all cases, the empirical Bayes model used to compare the two groups is first fitted to each subset of samples involving all features, then the filter is applied leaving out increasing fractions of the features. The power is estimated by comparing the proportion of differentially expressed features left in the data, out of all in the whole dataset (all samples and all features) declared differentially expressed.

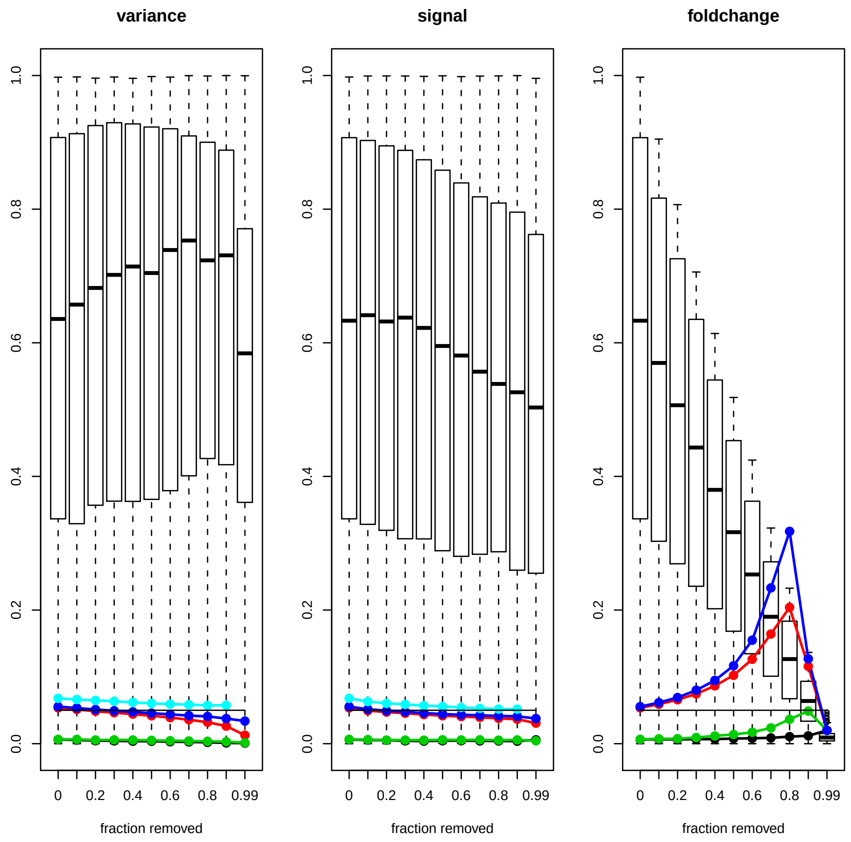

Figure 4: Results of our proposed FDR bias test, for the various filter statistics and FDR methods considered, and a range of filtered-out ratios (x-axis). For each filtered-out ratio, the empirical distribution of the null p-values is represented by a boxplot, and the p-value resulting from its comparison to the uniform is given above the graph: the p-value if not significant, '***' if less than 0.001. In all cases, the FDR was controlled at 0.05. Also shown are the lines representing the achieved FDR values, obtained from the non-permuted data using: BH (red), aBH (dark blue), qvalue (light blue) and BY (green). In addition, the Bonferroni correction is shown in black for comparison. A solid, thin black line, representing the FDR threshold is also shown. Results of the q-value method could only be obtained for the variance and signal filter, as for the fold change and test-statistic ones the p-value range was reduced, violating an assumption of the method.

Also the false positive rate behaviour can be examined, which corresponds to the proportion of the features declared differentially expressed in the subset under study but not in the whole dataset (all samples), divided by the total of features not declared significant in the whole dataset. As before, the fold change filter has the biggest effect, with the signal and variance filter remaining stable. As this represents the fraction of all null features that are wrongly seen as differentially expressed, it increases with the sample size due to the increasing number of features declared differentially expressed as the sample size increases. This fraction stabilizes and decreases again after a certain sample size threshold (data not shown).

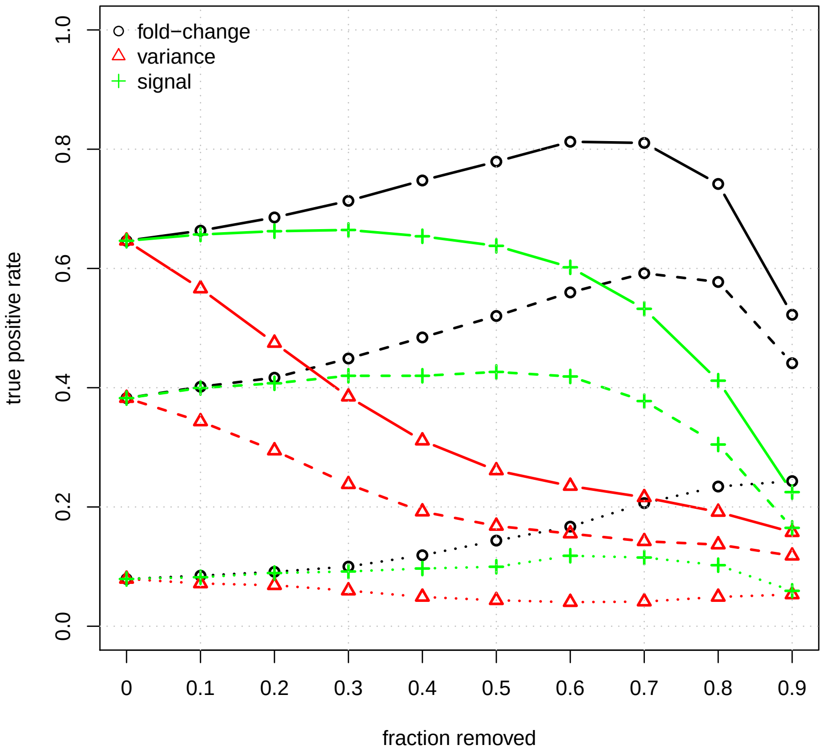

Figure 5: Achieved power (true positive rate) as function of the fraction filtered out for the different filter statistics, fixing the FDR with each method at 0.05. Computations are done using randomly selected subsets of $n = 8, 16, 24$ samples from each subtype considered. Differential expression is evaluated using limma, and p-values are FDR-corrected.

11 Performance of filtering using the Wilcoxon test

Our results hold for more general analysis models, in particular when another test is used other than the Student-t test. To show this, let us assume that instead the Wilcoxon rank-sums test was used. By re-running the simulation study achieved power and FDR can be evaluated as a function of the fraction of features filtered out, as before. For simplicity, we consider only BH as FDR correction. We can see from figure 6 that the obtained curves are very similar to those obtained using the Student's t-test (figures 5 of main text and 2 of this supplement).

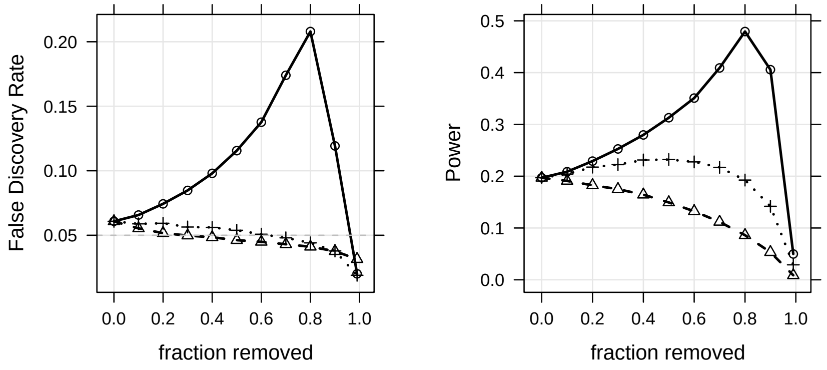

Figure 6: FDR (left) and actual power (right) as function of the fraction filtered out for the different filter statistics, fixing the FDR (Benjamini-Hochberg method) with each method at 0.05. Values shown are the mean FDR over 1000 simulated datasets (the variability of the FDR is small - not shown). Filters are: solid line = fold change, dashed = signal, dashed-and-dotted = variance, dotted = Wilcoxon-test statistic. In all cases the proportion of differentially expressed genes is fixed at 0.20.

12 FDR methods without uniform assumption for null p-values distribution

Here we have concluded that FDR methods that rely on the assumption that null p-values follow a uniform distribution can be biased if filtering is used prior to the statistical analysis. However, this could be circumvented by using another method to estimate the FDR that does not rely on this assumption.

Indeed, a number of such FDR methods has been proposed. One is Efron's local FDR, which assumes only that the test statistic distribution has a symmetric, unimodal distribution, and estimates the actual density empirically. Other methods that have been proposed include the empirical-Bayes and bootstrap-based FDR method of Dudoit and co-authors (hereafter bootFDR [9]), and the permutation-based methods of Jain and co-authors [10] and of Reiner and co-authors (hereafter permFDR [11]), the latter based upon previously suggested approaches which accomodated dependency [12] and used resampling [4]. While permFDR involves estimation and averaging of the p-values distribution over a number of permutations of the array labels, bootFDR involves both estimating the proportion of differentially expressed genes π_0 using empirical Bayes, as well as estimation of the null p-values distribution using bootstrap.

Efron's local FDR cannot in its current form be applied in conjunction with filters that yield a bimodal or asymetric null test statistic density, such as the fold-change filter. In addition, the control level of local FDR does not correspond to a unique control level of classic FDR, with Efron himself suggesting that either 10 or 20% control of the local FDR could correspond to control of the BH-FDR at 5%. For these reasons, the comparisons that follow exclude this method. In addition, the method proposed by Jain and

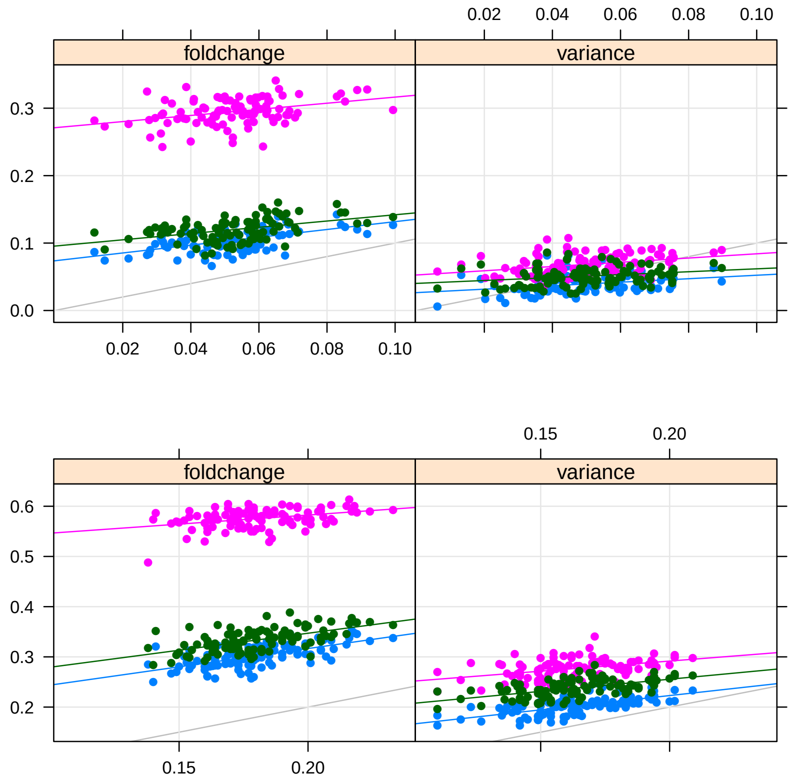

Figure 7: Relationship between achieved FDR (top row) and achieved power (bottom row) with filtering (y-axis) and without it (x-axis), for the fold change (left) and variance (right) filters. The light gray line represents $x = y$. Colours indicate the FDR estimation method used: BH-FDR (light blue), permFDR (dark green) and bootFDR (pink).

co-authors [10] is very similar to permFDR. So we will compare the classic FDR of Benjamini and Hochberg [3] (BH-FDR) with the permFDR of Reiner and co-authors [11] and with the bootFDR of Dudoit and co-authors [9].

As expected, for the variance filter the observed FDR with and without filtering are very similar, in contrast with results for the fold-change filter, which yields a biased FDR compared to when no filtering is used (top row of figure 7). With the fold-change filter, permFDR behaves very similarly to BH-FDR, yielding comparable amounts of FDR bias and of power improvement. On the other hand, bootFDR yields both FDR bias and power improvement compared to the other methods. This difference may be due to estimation of π_0, done using empirical Bayes by bootFDR but assumed to be 1 by BH-FDR and

permFDR. Another possible explanation is that all methods make assumptions in order to estimate the null p-values distribution, which are in their turn violated by the filtering. Furthermore, the null p-values distribution estimate is only correct for large samples and if either the sample sizes of both groups are equal, or the two groups have the same covariance structure, conditions which often do not hold in practice [13]. In practical applications, permutation-based null distribution estimates can also be biased if, for example, relevant covariates were not included in the model or in presence of correlation between sample and features [14]. Our conclusions are that even FDR estimation methods that use an empirical null p-values distribution may yield FDR bias, and that null p-values distribution estimation needs to be further studied in the context of filtering.

13 Use of filter after multiple testing correction

In this paper we have focussed on examining the effect of filtering prior to statistical analysis, and showed that some types of filter may introduce bias on commonly-used methods to estimate the FDR. In practice, researchers sometimes use filtering after multiple testing correction, typically to reduce a list of differentially expressed genes to only those more likely to be successfully validated in lab. In theory, there is no guarantee that the reduced list so produced will involve the same proportion of false positives as the full list generally. However, for some combinations of test statistic and post-FDR filter statistic the FDR may be little affected by the post-FDR filter, and it is unclear how the power is affected.

We use the same simulation study setup as before (section 8 of this Additional File), with 20% differentially expressed genes and mean differential expression levels generated at random from a bitriangular distribution. Also, as before the Student-t test is used to find differentially expressed genes, and genes are declared as differentially expressed if their FDR is up to 5%, using Benjamini-Hochberg's FDR step-up procedure [3]. We then consider three situations: (A) no filtering is used, (B) a fraction (25 or 50%) of the data is filtered prior to multiple testing correction, and (C) a fraction (10 or 20%) of the data is filtered out from the list of selected features, i.e., after multiple testing correction. In each situation, the observed FDR (the true proportion of false positives on the final list of selected genes) and the observed power (the true proportion of differentially expressed genes included in the final list) are computed.

The observed FDR (figure 8) is similar regardless of whether and when filtering is done for the variance and signal filters, whilst the fold change filter shows an FDR bias with pre-FDR filter but not with

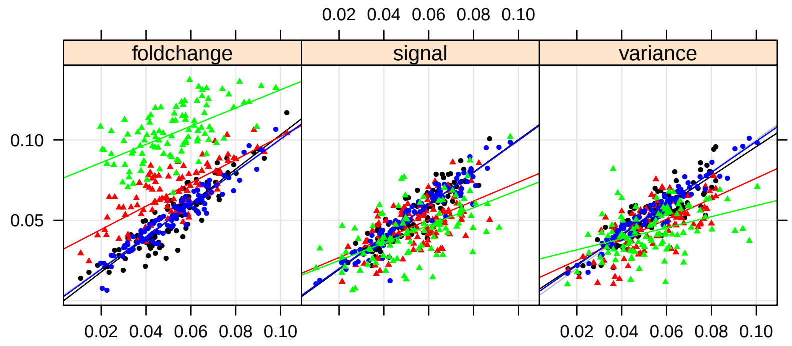

Figure 8: Comparison between observed FDR with no filtering (x-axis, situation A) with values observed with filtering (y-axis). In all cases the FDR was controlled at 5%. Blue and black dots: situation C with 10 and 20% left out, respectively; red and green triangles: situation B with 25 and 50% left out, respectively. The gray line represents $x = y$.

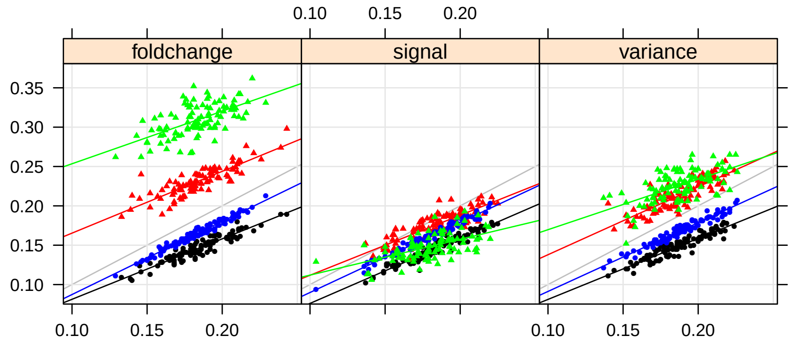

Figure 9: Comparison between observed power with no filtering (x-axis, situation A) with values observed with filtering (y-axis). Key for symbols and colours is given in figure 8.

post-FDR filter. The observed power (figure 9) reflects these observations in general, with the fold-change filter the only one to yield improved power with pre-FDR filtering. However, post-FDR filtering yields a slight loss of power in all cases. This leads us to conclude that post-FDR filtering does not bias the FDR and yields a small loss of power, in the setting used.

References

1. Finner H, Thorsten D, Roters M: **Dependency and false discovery rate: asymptotics**. *The Annals of Statistics* 2007, **35**(4):1432–1455.

2. Langaas M, Lindqvist B, Ferkingstad E: **Estimating the proportion of true null hypotheses, with application to DNA microarray data**. *Journal of the Royal Statistical Society Series B* 2005, **67**(4):555–572.

3. Benjamini Y, Hochberg Y: **Controlling the false discovery rate: a practical and powerful approach to multiple testing**. *Journal of the Royal Statistical Society Series B* 1995, **57**:289–300.

4. Benjamini Y, Yekutieli D: **The control of the false discovery rate in multiple testing under dependency**. *Annals of Statistics* 2001, **29**(4):1165–1188.

5. Benjamini Y, Krieger A, Yekutieli D: **Adaptive linear step-up procedures that control the false discovery rate**. *Biometrics* 2006, **93**(3):491–507.

6. Storey J: **A direct approach to false discovery rates.** *Journal of the Royal Statistical Society Series B* 2002, **64**:479–498.

7. R Development Core Team: *R: A Language and Environment for Statistical Computing.* R Foundation for Statistical Computing, Vienna, Austria 2007. [ISBN 3-900051-07-0].

8. Ferreira J, Zwinderman A: **On the Benjamini-Hochberg Method**. *The Annals of Statistics* 2006, **34**(4):1827–1849.

9. Dudoit S, Gilbert H, van der Laan M: **Resampling-based empirical Bayes multiple testing procedures for controlling generalized tail probability and expected value error rates: focus on the false discovery rate and simulation study**. *Biometrical Journal* 2008, **50**:716–744.

10. Jain N, Cho H, O'Connell M, Lee J: **Rank-invariant resampling based estimation of false discovery rate for analysis of small sample microarray data**. *BMC Bioinformatics* 2005, **6**:187.

11. Reiner A, Yekutieli D, Benjamini Y: **Identifying differentially expressed genes using false discovery rate controlling procedures.** *Bioinformatics* 2003, **19**(3):368–75.

12. Yekutieli D, Benjamini Y: **Resampling-based false discovery rate controlling multiple test procedures for correlated test statistics**. *Journal of Statistical Planning and Inference* 1999, **82**:171–196.

13. Pollard K, van der Laan M: **Choice of a null distribution in resampling-based multiple testing**. *Journal of Statistical Planning and Inference* 2004, **125**:85–100.

14. Efron B: **Microarrays, empirical Bayes and the two-groups model**. *Statistical Science* 2008, **23**:1–22.
